# Pulmonary valve myxoma requiring pulmonary valve replacement: a case report

**DOI:** 10.1186/s40792-022-01420-x

**Published:** 2022-04-14

**Authors:** Sawaka Tanabe, Keita Yano, Tae Mizunaga, Yuko Kawamura, Atsushi Takamori, Narihisa Yamada, Koichi Morioka, Takaaki Koshiji

**Affiliations:** Department of Cardiovascular Surgery, Faculty of Medical Sciences, University of Fukui, Fukui, 910-1193 Japan

**Keywords:** Cardiac tumor, Pulmonary valve myxoma, Right ventricular outflow tract tumor

## Abstract

**Background:**

Most cardiac myxomas occur in the atria. Myxomas arising from the heart valves are rare, and there are only a few reports of myxomas arising from the pulmonary valve. Complete resection and prevention of embolization at the time of the first surgery are important to prevent the recurrence of myxomas.

**Case presentation:**

An 82-year-old female was scheduled to undergo surgery for a fracture of the right femoral neck. The preoperative echocardiography showed a mass in the right ventricular outflow tract. The mass was 36 × 30 mm in size and entered into the pulmonary artery during systole. Cardiac synchronous computed tomography showed a stalked bifurcated mass near the pulmonary valve, which was suspected to be a myxoma. Surgical findings showed a lumen-occupying tumor when the main pulmonary artery was incised. Since the tumor was a single mass with a stalk on the pulmonary valve (right and left pulmonary valve cusps), tumor resection and pulmonary valve replacement (bioprosthetic valve) were performed. A right prosthetic femoral head insertion was performed on postoperative day 36, and the patient was transferred to the hospital on postoperative day 44. However, 1 year later, the patient developed a large myxoma (recurrence) that completely occluded the right pulmonary artery and died of right heart failure.

**Conclusions:**

We report the case of a patient with a very rare myxoma arising from the pulmonary valve, which was treated with tumor resection and pulmonary valve replacement surgery; however, the patient developed another myxoma 12 months later and this tumor was larger than the primary tumor. The surgical margins were indistinct, and there was a high possibility of residual tumor in the pulmonary artery wall; hence, an extended resection should have been considered. The recurrence of myxoma, in this case, suggests that it is important to completely resect the primary tumor during the first surgery and to prevent intraoperative embolization.

## Background

Of all cardiac myxomas 75% occur in the left atrium, 15–20% in the right atrium, and 3–4% in the ventricles [1, 2]. Myxomas rarely arise from the pulmonary valve. To the best of our knowledge, only eight cases, including case reports and autopsies, have been reported in the world [2–5]. We report a case of myxoma arising from the pulmonary valve in an elderly woman.

## Case presentation

An 82-year-old woman fractured her right femoral neck, following a fall; however, there was no loss of consciousness. She was scheduled for surgery for the treatment of right femoral fracture. The preoperative echocardiography showed a mass in the right ventricular outflow tract; hence, she was referred to our department. She had a history of hypertension and had undergone surgery for uterine fibroid. The patient was asymptomatic, with an oxygen saturation of 98% (room air) and no symptoms of heart failure, such as leg edema. Electrocardiography revealed sinus rhythm and chest radiography showed mild cardiac enlargement with a cardiothoracic ratio of 0.53.

**Fig. 1:**
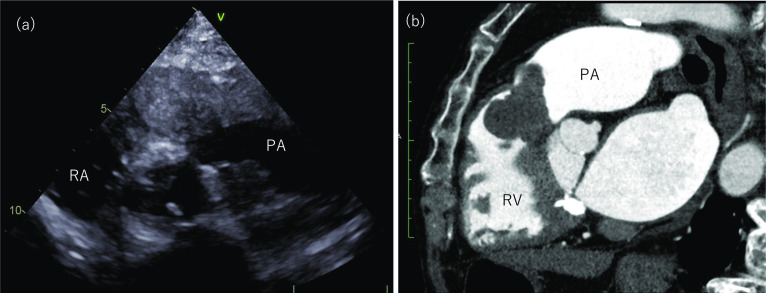
Preoperative imaging. **a** Transthoracic echocardiography shows a 36 × 30 mm mass in the right ventricular outflow tract. **b** Contrast-enhanced computed tomography of the chest shows a contrast defect around the pulmonary valve with a bifurcated morphology. There is minimal staining in the early contrast phase but slightly contrasted in the delayed phase. *PA, pulmonary artery; RA, right atrium; RV, right ventricle

Transthoracic echocardiography (TTE) revealed a 36 × 30 mm mass in the right ventricular outflow tract, mild tricuspid regurgitation with a pressure gradient of 51 mmHg, and no enlargement of the right ventricle or right atrium (Fig. 1a).

Contrast-enhanced computed tomography (CT) revealed a contrast defect around the pulmonary valve with a bifurcated morphology. The early contrast phase did not stain well; however, the delayed phase was slightly contrasted (Fig. 1b).

Coronary CT showed no significant stenosis in the coronary arteries.

Cardiac magnetic resonance imaging revealed a stalked mass in the pulmonary valve with mixed high and moderate signals on T2.

**Fig. 2:**
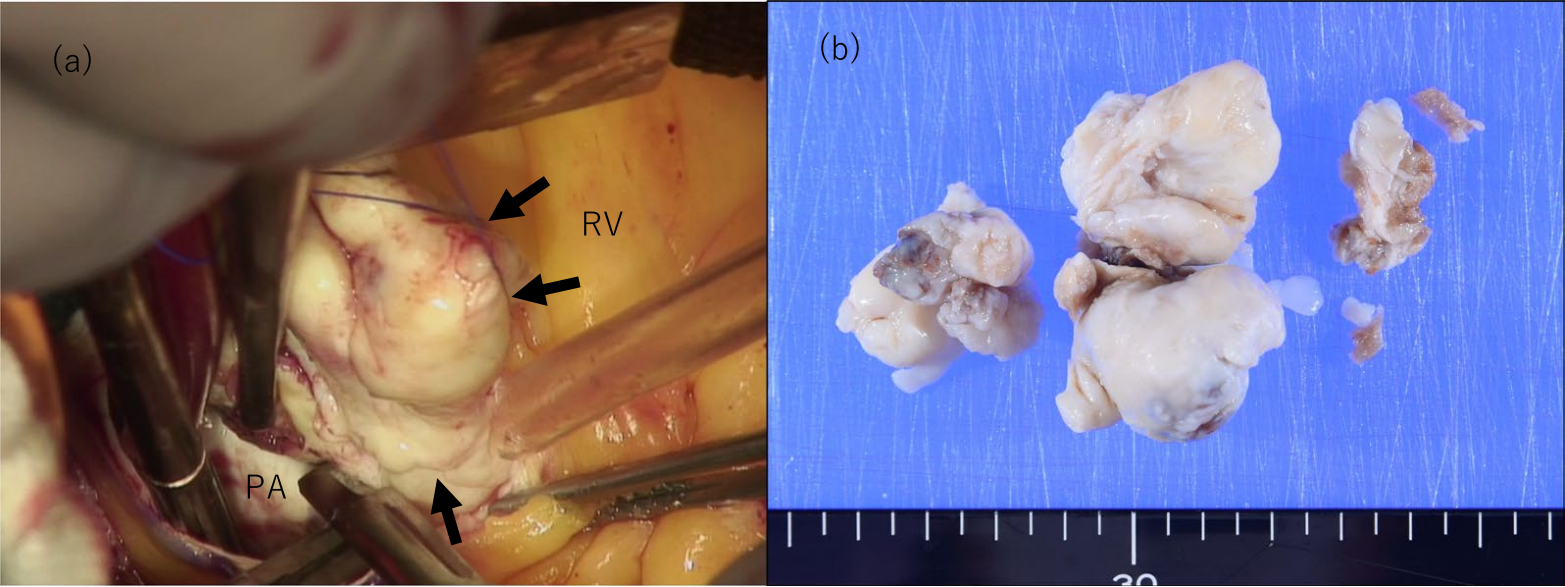
Surgical findings. **a** Intraoperatively, the tumor (black arrow) is firmly lumped with the pulmonary valves and also partially extends to the posterior wall of the pulmonary artery. **b** Surgical specimen

The patient was operated on total cardiopulmonary bypass and the tumor was resected. Surgical findings showed that the lumen of the main pulmonary artery, accessed through a longitudinal incision, was occupied by the tumor. All 3 cusps were lumped together with the tumor, especially in the area of the left and right pulmonary artery valves. The tumor was resected and a 21-mm CEP Magna (Edwards Lifesciences, Irvine, CA, USA) was placed in the valve ring. The tumor partially extended to the posterior wall, and upon resection, a small hole was found in the posterior wall, which was repaired (Fig. 2a).

**Fig. 3:**
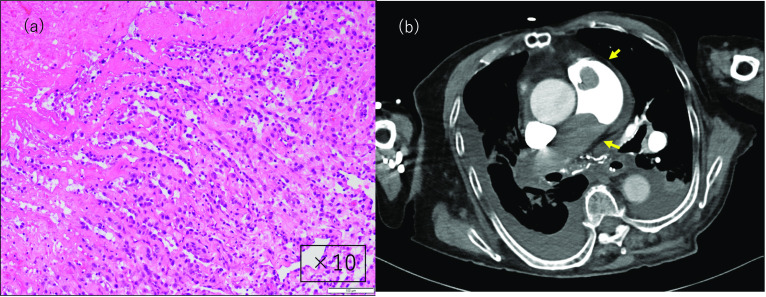
Pathological findings and follow-up. **a** Pathological examination findings reveal a white, substantial, mass lesion. Histology shows keloid-like proliferation of mature collagen fibers. Although the lesion is obsolete, there is a possibility of a myxoma in the background. Immunostaining is not suggestive of malignancy. **b** Contrast-enhanced computed tomography at recurrence 1 year later shows a tumor (yellow arrow) in the pulmonary valve (prosthetic valve) and pulmonary artery

Pathologic examination revealed a white, substantial mass lesion. Histology showed keloid-like proliferation of mature collagen fibers. Immunostaining did not reveal the presence of calretinin-positive cells, which was atypical; however, based on hematoxylin–eosin staining, it was diagnosed as a myxoma with secondary changes. The images were not suggestive of malignant transformation (Figs. 2b and 3a).

The postoperative course was uncomplicated. The patient underwent right artificial femoral head insertion on postoperative day 36 and was transferred for rehabilitation on postoperative day 44. Six months after surgery, echocardiography showed no recurrence. However, 1 year later, the patient was re-examined for respiratory symptoms, and myxoma recurrence was seen in the pulmonary valve (artificial valve) and pulmonary artery. Due to her poor general condition, she was unfit to undergo surgery and died of right heart failure (Fig. 3b).

## Discussion

The majority of the cardiac myxomas originate in the atrial wall; ventricular and heart valve myxomas are less common. Myxomas arising from the pulmonary valve are particularly rare. Until now, only eight cases have been reported worldwide through case reports and autopsies [2–5]. Therefore, there is a lack of information about the age distribution, surgical procedure, and recurrence of these myxomas.

Myxomas of the pulmonary valve and pulmonary arteries are thought to arise in situ or as metastases from distant myxomas [6]. In our case, no myxoma was found at any location other than the primary tumor in the pulmonary valve, and it was considered to be a myxoma originating from the pulmonary valve.

The general complications of myxomas include hemodynamic obstruction, embolization, or constitutional changes. The most common complications of myxoma include systemic embolization of the cerebral arteries, renal arteries, and aorta; pulmonary embolism and pulmonary hypertension; symptoms of cardiac obstruction, such as heart failure, syncope, mitral and tricuspid valve insufficiency, and sudden death; and systemic signs and symptoms, such as malaise, anorexia, fever, arthralgia, anemia, weight loss, and increased levels of C-reactive protein and globulin [1].

Symptoms specific to right ventricular outflow tract tumors include syncope, arrhythmia, pulmonary embolism, valve dysfunction, and sudden death [7, 8]. The symptoms of the pulmonary valve and pulmonary artery myxomas are similar to those of right ventricular outflow tract tumors. Pulmonary valve myxoma might be misdiagnosed as pulmonary artery embolus, thrombus, or verruca, leading to inappropriate treatment with anticoagulants or thrombolytics [5, 9]. Other differential diagnoses include sarcoma and metastatic tumors [10].

The treatment for myxoma is surgical resection; however, the recurrence rate is reported to be 13% at 10 years [11]. Nevertheless, the number of cases of pulmonary valve myxoma is small, and the long-term prognosis is unknown [5].

Reynen suggested the transition from benign to a malignant tumor as an explanation for the recurrence of myxoma [1]. In this case, the myxoma recurred in the pulmonary artery 1 year later. Unfortunately, the patient was already in a very poor respiratory state at the time of recurrence, and a biopsy could not be performed. An autopsy was not conducted respecting the wishes of the family. Therefore, it was not possible to confirm whether the recurrence was due to malignant transformation. Kabbani suggested that the cause of recurrence might be local cytotransplantation of the primary tumor [12]. Therefore, Kabbani recommends a biatrial approach for intracardiac myxomas to ensure resection of the atrial wall, including the tumor, and to prevent intraoperative embolization. Read also suggested that recurrence is caused by systemic embolization of myxoma cells [13]. Furthermore, Read reported that recurrent cardiac myxomas grow more rapidly than primary tumors, and it is important to completely resect the primary tumor during the first surgery. In this case, the tumor partially extended to the posterior wall where a small hole was made during resection. The surgical margins were indistinct, and there was a high possibility of residual tumor in the pulmonary artery wall; hence, an extended resection should have been considered. However, the patient’s condition was highly fragile, and she was unable to walk after fracturing her right femur. She could not tolerate a highly invasive surgery; hence, we chose to perform valve replacement surgery. However, right ventricular outflow tract reconstruction could have been performed to prevent recurrence. The recurrence in this case also suggests that intraoperative embolization may not have been adequately considered. Embolization should have been firmly blocked using gauze or other means during tumor resection to prevent the embolization from falling into the peripheral pulmonary artery.

In general, cardiac myxomas tend to occur in the atrial wall [1], and there are only a few reports of their occurrence in the heart valve itself. Mitral, tricuspid, and aortic valves account for the majority of reports of myxomas occurring in heart valves, for which valvuloplasty or valve replacement is the procedure of choice, depending on the degree of valve destruction [14–19]. Reports of myxomas occurring in the pulmonary artery valve are rare. Therefore, there is a lack of information about the operative technique and prognosis; however, our experience suggests that complete resection at the time of initial surgery and intraoperative embolic prophylaxis are important to prevent a recurrence.

## Conclusion

We encountered a rare case of myxoma arising from the pulmonary valve. The myxoma was associated with pulmonary valve destruction, and valve replacement was performed in addition to tumor resection; however, the tumor recurred 1 year later. At the time of recurrence, the myxoma was larger than the primary tumor.

## Data Availability

Data sharing is not applicable for this article as no datasets were generated or analyzed during the current study.

## Abbreviations

CT: Computed tomography
TTE: Transthoracic echocardiography
